# Genomics in aquaculture to better understand species biology and accelerate genetic progress

**DOI:** 10.3389/fgene.2015.00128

**Published:** 2015-04-01

**Authors:** José M. Yáñez, Scott Newman, Ross D. Houston

**Affiliations:** ^1^Faculty of Veterinary and Animal Sciences, University of ChileSantiago, Chile; ^2^AquainnovoPuerto Montt, Chile; ^3^Genus plcHendersonville, TN, USA; ^4^The Roslin Institute and Royal (Dick) School of Veterinary Studies, University of EdinburghMidlothian, UK

**Keywords:** aquaculture, genome, breeding programs, QTL, single nucleotide polymorphisms, next-generation sequencing

The production of fish and shellfish through aquaculture is an increasingly important source of high-quality animal protein, with a worldwide production of 66.6 million tons in 2012 (FAO, 2014). Considering the continuously growing global human population and increasing demand for fish products, improvements in the scale, efficiency, and sustainability of aquaculture are essential. To achieve this, several challenges facing the culture of fish and shellfish species need to be overcome. These relate to the diverse biology of the cultured species and their interaction with environmental factors. Examples include outbreaks of infectious diseases, control of sexual maturation, sustainable feed for carnivorous species, and tolerance of diverse and changing environments. This “Frontiers in Livestock Genomics” Research Topic highlights the opportunities offered by recent developments in the field of genomics, and in particular high-throughput sequencing, to contribute to addressing these challenges, with a focus on selective breeding programmes.

The use of selective breeding as a tool to improve the biological efficiency of production in aquaculture generally lags behind plant and farm animal industries, and less than 10% of aquaculture production is based on genetically-improved stocks (Gjedrem et al., 2012). Encouragingly, annual genetic gains reported for aquatic species are in general substantially higher than that of terrestrial farm animals (Gjedrem et al., 2012) and there is considerable scope for achieving significant positive economic impact via improved breeding schemes. However, the status of breeding programs and the level of technology used for aquatic species production are wide-ranging, from use of wild seed stocks through to family-based selection incorporating genomic tools. Family selection and genomic tools can be applied to improve traits that are expensive or difficult to measure on the selection candidates themselves including disease resistance (Yáñez et al., 2014; Ødegård et al., 2014), flesh color (Colihueque and Araneda, 2014; Ødegård et al., 2014) and other appearance traits such as body shape and skin pigmentation (Colihueque and Araneda, 2014) in finfish species. In contrast, despite the global importance of mollusc species for aquaculture, few selective breeding programmes exist and the state of genomic tools and knowledge for these species is typically lacking (Astorga, 2014).

Genomics resources such as whole genome reference sequences, high-density SNP genotyping arrays and genotyping-by-sequencing are in development for several aquaculture species. Fuller characterisation of these resources is underway and is resulting in improved fundamental knowledge of the genome structure and biology, highlighted in this issue by the analysis of repeat elements in the Asian sea bass genome (Kuznetsova et al., 2014). These resources will provide powerful tools for the research community and will aid in the determination of the genetic factors involved in the regulation of complex traits. For example, high-throughput RNA sequencing can give a holistic view of the host response to infectious diseases, and help identify the important genes and pathways defining genetic resistance, as demonstrated in this issue for rainbow trout (Ali et al., 2014; Marancik et al., 2014) and panaeid shrimp (Santos et al., 2014). Sequencing technology has also facilitated the development of abundant genetic markers that have multi-faceted applications for selective breeding of aquatic species, including parentage assignment in mixed-family environments, providing greater control over family representation and inbreeding (Vandeputte and Haffray, 2014). Medium or high-density SNP arrays can be used to predict genomic breeding values for economically-important traits in well-developed breeding programmes, such as Atlantic salmon (Ødegård et al., 2014). For instance, based on simulations of a Pacific white shrimp breeding program, genetic progress of disease resistance traits is faster with genomic-enabled selection compared to conventional phenotype-based selection due to higher accuracy (Castillo-Juárez et al., 2015). Incorporation of genetic marker information can also be a useful asset to optimize genetic diversity and future genetic gain when establishing base populations for breeding programmes (Fernández et al., 2014). Furthermore, these genomic tools can be applied to investigate putative genomic signatures of selection during the domestication process of farmed fish species, thus potentially identifying genomic regions underlying variation in relevant phenotypes in wild and domestic fish populations (López et al., 2015).

Aquaculture species typically have several common features, for example high fecundity and external fertilization, plus a short evolutionary distance from their wild ancestors. The reproductive features enable flexible mating structures to be used for breeding programmes, and can provide a powerful resource for genetic studies of complex traits, such as disease resistance (Yáñez et al., 2014). However, the diversity between these species is enormous and often necessitates the establishment of species-specific reproduction and breeding programmes. For example, there is a remarkable variety of sex-determination systems within aquatic farmed species, and the study of Martínez et al. (2014) highlights various methods of controlling sex ratio with aquaculture breeding programmes. This species diversity also presents an issue for choosing suitable model organisms to inform on the biology of the farmed species of interest. Model finfish species, such as zebrafish, have been well-characterized and Ulloa et al. (2014) highlight their utility for the evaluation of the response to alternative diets. However, due to the vast evolutionary distance between certain farmed aquatic and model species, it is clear that direct research on the species of interest can often be the most feasible and informative.

The aquaculture industry has often been innovative and visionary in their application of new technologies to improve production. Genomics present another major opportunity, and the research published in this special issue provides several excellent examples of their potential or realized application. Using genomic tools to more effectively utilize genetic variation in economically-important traits via sustainable breeding programmes is paramount to the continued successful growth and stability of aquaculture production.

## Conflict of interest statement

The authors declare that the research was conducted in the absence of any commercial or financial relationships that could be construed as a potential conflict of interest.
